# Housefly Population Density Correlates with Shigellosis among Children in Mirzapur, Bangladesh: A Time Series Analysis

**DOI:** 10.1371/journal.pntd.0002280

**Published:** 2013-06-20

**Authors:** Tamer H. Farag, Abu S. Faruque, Yukun Wu, Sumon K. Das, Anowar Hossain, Shahnawaz Ahmed, Dilruba Ahmed, Dilruba Nasrin, Karen L. Kotloff, Sandra Panchilangam, James P. Nataro, Dani Cohen, William C. Blackwelder, Myron M. Levine

**Affiliations:** 1 Center for Vaccine Development, School of Medicine, University of Maryland, Baltimore, Maryland, United States of America; 2 International Centre for Diarrhoeal Disease Research (ICDDR,B), Dhaka, Bangladesh; 3 Department of Pediatrics, School of Medicine, University of Virginia, Charlottesville, Virginia, United States of America; 4 School of Public Health, Sackler Faculty of Medicine, Tel Aviv University, Ramat Aviv, Israel; The Johns Hopkins University, United States of America

## Abstract

**Background:**

*Shigella* infections are a public health problem in developing and transitional countries because of high transmissibility, severity of clinical disease, widespread antibiotic resistance and lack of a licensed vaccine. Whereas *Shigellae* are known to be transmitted primarily by direct fecal-oral contact and less commonly by contaminated food and water, the role of the housefly *Musca domestica* as a mechanical vector of transmission is less appreciated. We sought to assess the contribution of houseflies to *Shigella*-associated moderate-to-severe diarrhea (MSD) among children less than five years old in Mirzapur, Bangladesh, a site where shigellosis is hyperendemic, and to model the potential impact of a housefly control intervention.

**Methods:**

Stool samples from 843 children presenting to Kumudini Hospital during 2009–2010 with new episodes of MSD (diarrhea accompanied by dehydration, dysentery or hospitalization) were analyzed. Housefly density was measured twice weekly in six randomly selected sentinel households. Poisson time series regression was performed and autoregression-adjusted attributable fractions (AFs) were calculated using the Bruzzi method, with standard errors via jackknife procedure.

**Findings:**

Dramatic springtime peaks in housefly density in 2009 and 2010 were followed one to two months later by peaks of *Shigella*-associated MSD among toddlers and pre-school children. Poisson time series regression showed that housefly density was associated with *Shigella* cases at three lags (six weeks) (Incidence Rate Ratio = 1.39 [95% CI: 1.23 to 1.58] for each log increase in fly count), an association that was not confounded by ambient air temperature. Autocorrelation-adjusted AF calculations showed that a housefly control intervention could have prevented approximately 37% of the *Shigella* cases over the study period.

**Interpretation:**

Houseflies may play an important role in the seasonal transmission of *Shigella* in some developing country ecologies. Interventions to control houseflies should be evaluated as possible additions to the public health arsenal to diminish *Shigella* (and perhaps other causes of) diarrheal infection.

## Author Summary

Whereas previous researchers have noted that seasonal peaks in the numbers of houseflies and patients suffering from *Shigella* diarrheal infection seemed to coincide, this is the first research to quantify the association using time-series statistical methods. The results show that houseflies could account for approximately 37% of all cases of shigellosis in an area in rural Bangladesh. This research adds to the existing published experimental and observational evidence from other parts of the world implicating houseflies as mechanical transmission vectors for *Shigella*. The results can be used to advocate for cluster-randomized intervention trials that can demonstrate how much control of housefly density can diminish *Shigella* disease incidence. This question should be answered because there are currently no licensed *Shigella* vaccines, and rising antibiotic resistance is limiting treatment options. Control of houseflies using methods such as baited fly traps could be an affordable, effective intervention to add to the public health arsenal for routine use and in the context of disaster response.

## Introduction

*Shigella*, a human host-restricted pathogen that invades and damages gut mucosa, persists as a public health problem in developing and transitional countries because of its high transmissibility via direct fecal-oral contact, the severe clinical disease it causes, widespread drug resistance that limits the utility of previously effective antibiotics and the absence of licensed vaccines. The minute inoculum (ten *Shigella* organisms) capable of causing full blown dysentery enables direct person-to-person transmission [1], [2], even where environmental sanitation is otherwise adequate and safe water is available [3], [4]. Less commonly, *Shigella* is transmitted by contaminated food [5] or water vehicles [5]. Least appreciated is the observational and robust experimental evidence that demonstrates that the housefly, *Musca domestica*, can serve as a mechanical vector that also fosters transmission of *Shigella* [6], [7].

Houseflies breed in human feces [8], *Shigella* can be cultured from flies trapped in endemic areas [6], [9], [10], and observational studies have shown increased incidence of dysentery or diarrhea during periods of high fly density [11]–[13]. Most importantly, controlled intervention studies have shown that reducing housefly density is accompanied by reduced incidence of diarrhea [6], [11], [14], [15], dysentery [6], culture-confirmed shigellosis [6], [14], [15] and serological evidence of *Shigella* infection [6]. To gather evidence of the association of housefly population density with *Shigella*-associated illness among children <five years of age in a developing country setting, we systematically enumerated houseflies in sentinel households in Mirzapur, Bangladesh, a site characterized by an unusually high prevalence of *Shigella* among children with acute moderate-to-severe diarrhea (MSD), and few apparent risk factors for transmission of diarrheal disease pathogens, when compared with the other six sites in the Global Enteric Multicenter Study (GEMS) [16]. To our knowledge, this is the first study to attempt to correlate the density of houseflies in environs of typical households with the occurrence of laboratory-confirmed *Shigella*-associated illness in young children in the community.

## Methods

### Design

A cross-sectional study examining the association between site-wide housefly population density and *Shigella*-associated MSD among children <five years of age was carried out from December 3^rd^, 2008 to December 1^st^, 2010 in Mirzapur, Bangladesh.

### Ethics statement

The study was nested within the three-year GEMS, which included a matched case-control study of the burden and etiology of MSD. Informed consent was sought from parents or caretakers of the research subjects, all of whom were children <5 years of age. Study purpose, risks and benefits were first explained to caretakers of children invited to participate in GEMS before the consent form was read aloud, while the caretaker, if literate, read his or her own copy of the consent form. Ample time was allowed for questions and discussion. If the parent/caretaker consented, he or she was then asked to provide written consent by signing the consent form. If the caretaker was illiterate, a person not employed by the study was asked to witness the informed consent process; upon consent, both the caretaker and witness were asked to sign their names to the consent form (illiterate caretakers unable to provide a written signature were asked to apply an ink fingerprint impression instead). The presence of a witness signature indicated that consent was oral rather than written. Permission was obtained from the head of household for placement within the household compound of devices (Scudder grills) to quantify fly density. The consent forms and protocol, including the provision for oral consent, were approved by the ICDDR,B Ethical Review Committee and the University of Maryland Human Research Protections Office.

### Setting and study sample

Mirzapur is a mainly Muslim rural community 70 km northwest of Dhaka with a population of approximately 254,751 (∼24,077 children <five years of age) under a Demographic Surveillance System (DSS). Most men are engaged in agriculture or daily wage labor and women typically work in the home. Many households have one or more family members working long-term abroad (mainly in Persian Gulf States and Saudi Arabia) who send home financial supplements that substantially improve the household's economic situation. “Winter” generally lasts from December to mid-February, while the monsoon rains and flooding occur during the hot months of June to October. The months of March to May are warm and dry. Children 0–59 months of age living within the Mirzapur DSS area and presenting for care at Kumudini Hospital were registered, and those with diarrhea (≥three abnormally loose stools within the previous 24 hours) were screened for disease severity. MSD is defined as diarrheal illness of <seven days duration accompanied by clinical signs of moderate or severe dehydration (sunken eyes, loss of skin turgor) or administration of intravenous fluids based on clinical assessment, dysentery (blood visible in loose stools), or hospitalization based on clinical judgment [17]. Caretakers of children with MSD were invited to enroll their children in GEMS. Up to approximately nine MSD cases were enrolled per fortnight (though more may have presented) in each of three age groups: 0–11, 12–23 and 24–59 months [17]. Stool samples were examined for a wide array of bacterial, viral and protozoal pathogens [18].

### Microbiology

*Shigella* was identified by culture on differential and selective media [18].

### Measurement of housefly population density

**Figure 1:**
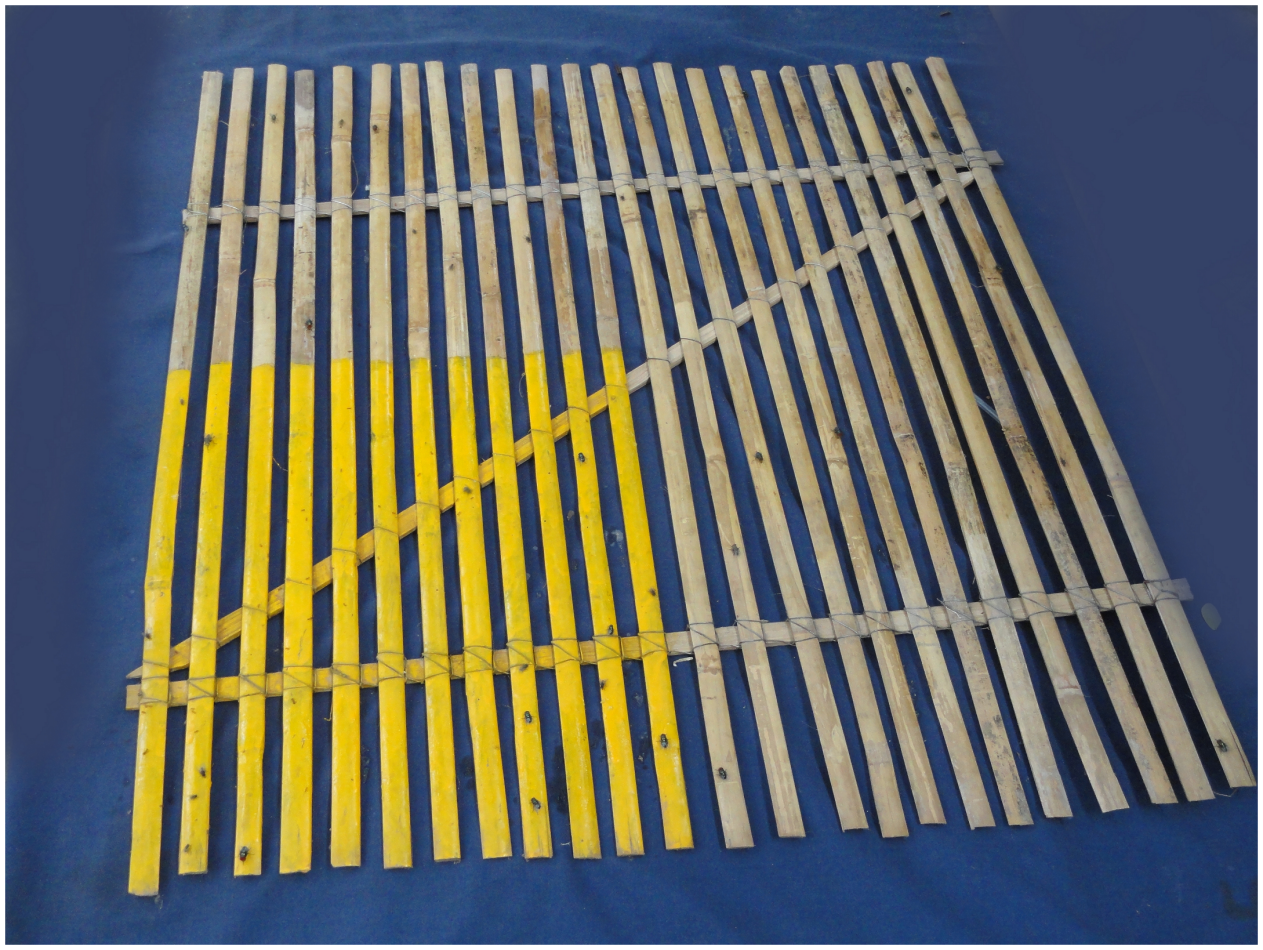
Scudder grill housefly enumeration device in use in Mirzapur.

**Figure 2:**
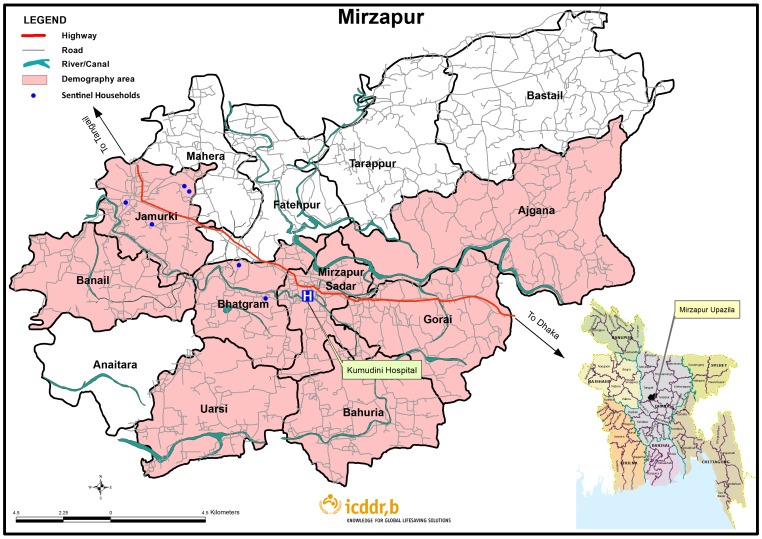
Map of the Mirzapur DSS showing Kumudini hospital and the 6 randomly-chosen sentinel households.

Houseflies were counted using a Scudder grill device (slats of wood screwed onto a Z-shaped wooden template to create a lattice), allowing counting in a standardized manner as houseflies typically alight on edges (Figure 1) [19]. One fourth of the Scudder grill was painted yellow to allow the flies to stand out visually; the restricted area allowed more practical counting when fly densities were high. The number of flies on the yellow area was multiplied by 4 to obtain a count for the entire grill. The Scudder grills were placed twice–weekly between 11 am and 2 pm in six sentinel household compounds selected at random from the DSS; grills were put near the household's latrine(s) or in cooking/eating areas where people and flies congregate and where there might be opportunities for mechanical contamination of food and eating utensils. Because the households were selected randomly, they tended to be clustered among the most densely populated area of the DSS (Figure 2). After field workers placed the Scudder grills on the ground or another flat surface, they waited for 30 minutes for flies to settle before counting.

### Temperature

Daily mean, maximum and minimum temperatures were obtained for the study period from the Dhaka weather station, approximately 70km away [20].

### Data analysis

Twice-weekly fly counts at all six sites were pooled to provide a Mirzapur-wide weekly count. For exploratory data analysis, weekly counts were summed and divided by the number of weeks falling primarily in a month to provide a mean weekly fly count for each month. For time series analysis, mean weekly fly counts that corresponded to the GEMS biweekly periods were summed and then divided by two. To calculate the estimated total number of children with *Shigella*-associated MSD presenting to Kumudini Hospital, the proportion of enrolled children testing positive was multiplied by the total number of eligible children presenting during that period. The biweekly average was calculated for each of the three daily temperature statistics, yielding an average mean, average maximum and average minimum temperature for each biweekly period, henceforth referred to simply as mean, maximum and minimum temperature.

### GLM Poisson time series regression

A transitional regression model (TRM) for autocorrelated count data was used for the primary analysis, with housefly population density as the explanatory variable and *Shigella*-associated case counts as the outcome [21]. The TRM is a generalized linear model (GLM) of the Poisson family, with a log link, in which autocorrelation is accounted for by including one or more lagged values of the outcome among the explanatory variables. The scale (whether untransformed or logarithmic) of the fly counts and the number of lags to include was determined by regressing all combinations of lags and minimizing Akaike Information Criterion (AIC) [22] and Bayesian Information Criterion (BIC) values [23] calculated using the *estat ic* command in Stata 12 (StataCorp, College Station, TX). To assess the possibility of a lagged effect of housefly population density on presentation of *Shigella*-positive cases, the housefly counts were lagged by one to seven biweekly periods, and AIC and BIC values were calculated to determine whether each lag (or combinations thereof) improved the model fit. To assess for the the possibility that temperature may be confounding the association between the housefly population density exposure and *Shigella* case count outcome, mean temperature was added to the model at one to seven lags, and the beta for log housefly population density was observed for a change >10% that would suggest confounding. Scatterplots of log *Shigella*-positive case counts on all lags of fly values and temperature in both untransformed and logarithmic scales were used to determine the appropriate scale.

All statistical analyses were performed using Stata 12.

### Autocorrelation-adjusted attributable fraction

To estimate the number of *Shigella* cases that could have been prevented by a public health intervention if flies were reduced to the level observed in the lowest 10% of biweekly periods, we used the Poisson regression output to calculate an attributable fraction (AF) that was adjusted for autocorrelation using the method originally developed by Bruzzi for adjusting for confounders [24], [25]. To enable the calculation, the fly count variable was converted into a decile, then regressed against *Shigella*-associated MSD case counts. A separate incidence rate ratio (IRR) was calculated for each decile (using the lowest decile as the referent), and was then used to estimate the percentage of infected cases that was attributable to flies. For each decile, this percentage was then multiplied by the total number of *Shigella*-associated cases to estimate the number of cases attributable to flies. These numbers of attributable cases were summed over the upper nine deciles, then divided by the total number of cases to estimate the AF. The standard error was calculated using a jackknife procedure [25], [26]. This procedure was repeated to estimate the AF of reducing flies to the level observed in the lowest 30% and 50% of periods by setting the referent to the lowest 3 deciles and lowest 5 deciles, respectively. Only the 50 periods on which the lagged effect might operate were counted in the denominator. The total number of *Shigella* cases observed during the 50 periods was 362.6.

## Results

### Housefly population density

**Figure 3:**
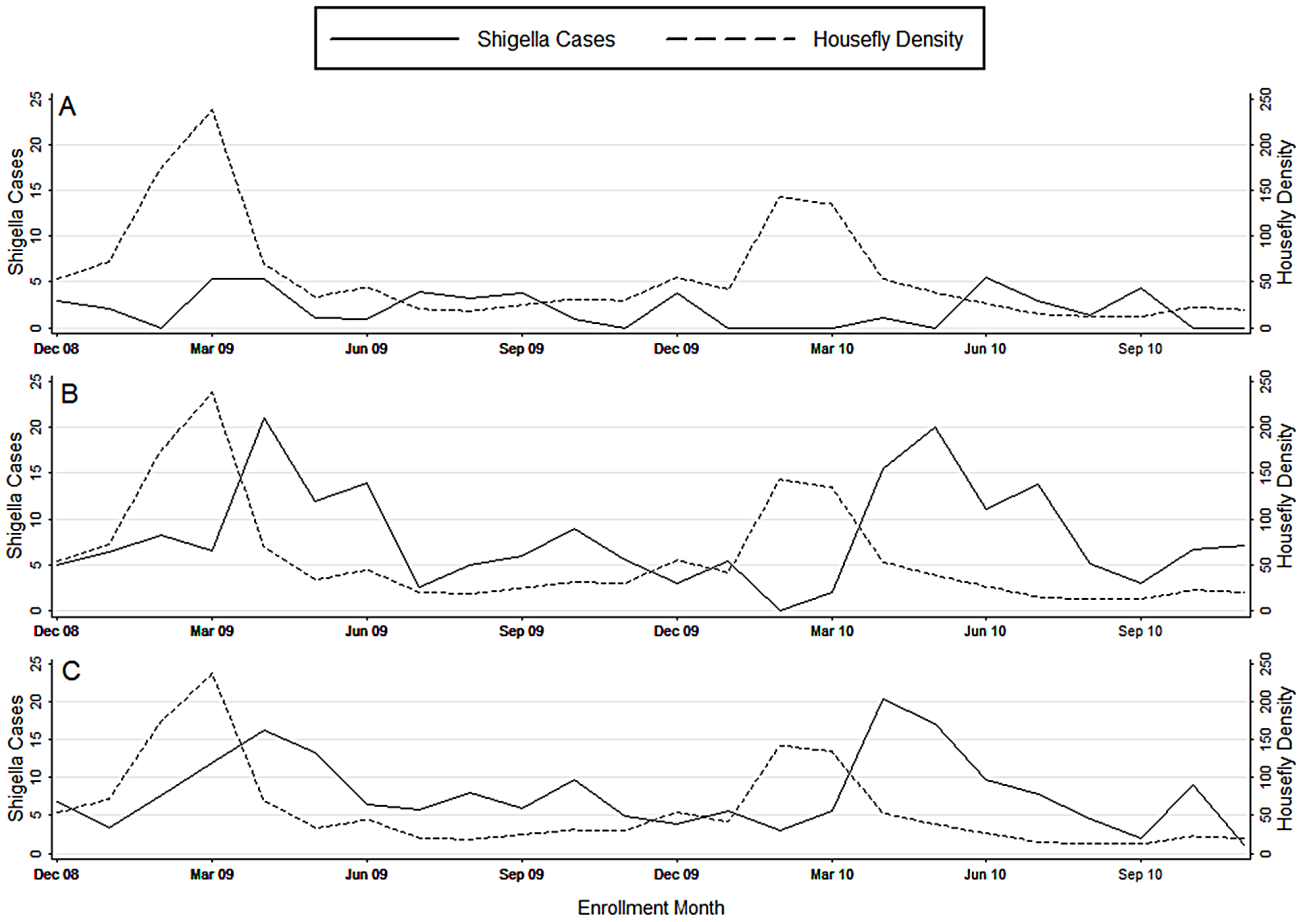
Mean weekly fly count and *Shigella*-associated cases presenting to Kumudini Hospital by calendar month. Children 0–11 (A), 12–23 (B) and 24–59 (C) months of age.

The study covered 53 biweekly periods of GEMS study enrollment. Housefly population density was stable, with the exception of two dramatic peaks that occurred in the late winter to early spring of 2009, and again in 2010 (Figure 3). In February 2009, fly density more than doubled from the previous month, rising to 174 flies/week, and climbed to 238 flies/week in March, before decreasing again to 70 flies/week. The following year, fly density more than tripled, rising from 42 to 143 flies/week in February and 135 flies/week in March before decreasing to 53 flies/week the following month.

### *Shigella*

Table 1 shows for each age group the number of children with MSD presenting to Kumudini Hospital, the number enrolled into the study, the number and percent positive for *Shigella*, and the estimated total number of *Shigella* cases (calculated by multiplying the percent *Shigella*-positive among enrolled by the total MSD cases). Among 391 children 0–11 months of age, there were relatively few *Shigella*-associated MSD cases (N = 40) and no obvious seasonal pattern (Figure 3A). By contrast, among the 343 toddlers 12–23 months of age, there was a large number of *Shigella*-associated MSD cases (N = 194) (Table 1) and a clear pattern showing spikes during the months of April, May and June of both 2009 and 2010 (Figure 3B). Similarly, among the 282 pre-school children 24–59 months of age, there was also a high number of both confirmed and estimated *Shigella*-associated MSD cases (N = 190) (Table 1) and an obvious pattern showing spikes of shigellosis in March, April and May of 2009 and April and May of 2010 (Figure 3C). The distribution of *Shigella* species included *S. flexneri* isolated from 224 children, *S. sonnei* from 108, *S. boydii* from 16 and *S. dysenteriae* from nine; there were five instances of dual infections between *S. flexneri* and species. There was no apparent association between a particular *Shigella* species and houseflies.

**Table 1:** Number estimated positive for MSD among all eligible children with MSD presenting at Kumudini Hospital.

Age group (months)	No. of eligible children presenting with MSD	No. enrolled	Culture-positive for *Shigella, n(%)*	Estimated total *Shigella* cases *
0–11	391	328	40 (12)	49
12–23	343	286	160 (56)	194
24–59	282	229	154 (67)	190

* Calculated by multiplying the percent positive enrolled MSD cases by the total MSD cases.

**Figure 4:**
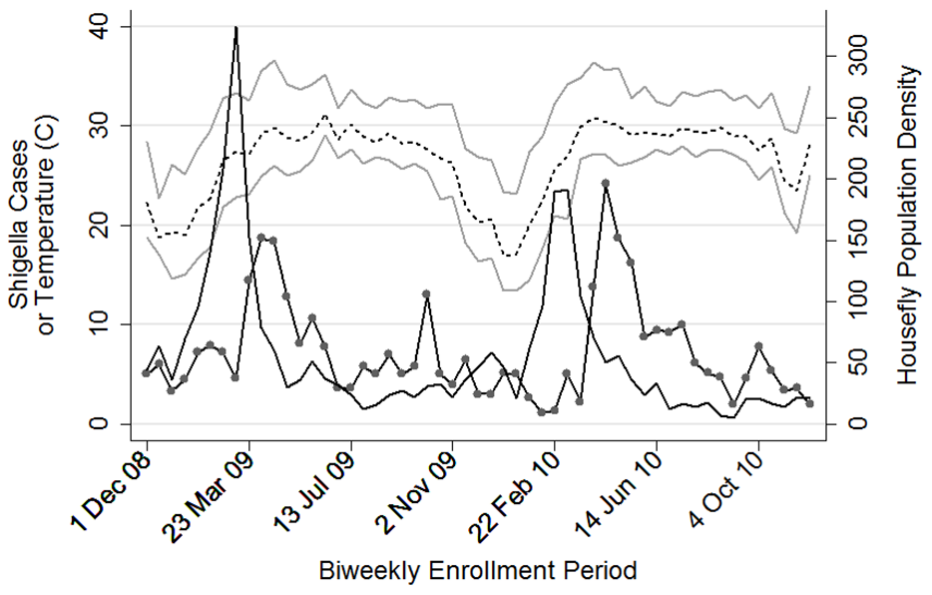
Mean weekly fly count (solid black line), *Shigella* cases (solid black line with circle connectors) and mean, maximum and minimum temperatures by biweekly enrollment period. Children 12–59 months of age. Average daily mean temperature (dashed line) with average daily maximum and minimum temperatures above and below (gray lines) in °C.

For the time series analysis, the 12–23 and 24–59 month age groups were combined to enable analysis of biweekly data, thus optimizing the sample size (number of time periods) for the Poisson model, while ensuring that there were enough cases in each period to avoid a zero-inflated data situation. This analysis showed a similar pattern compared with the monthly data and revealed that *Shigella* cases can vary by large amounts on a biweekly basis (Figure 4).

### *Shigella* and housefly population density

There was no apparent association between housefly population density and *Shigella*-associated MSD presentations among infants 0–11 months of age (Figure 3A). However, among toddlers 12–23 months of age, each spike in housefly population density was followed approximately two months later by a surge in *Shigella*-associated MSD (Figure 3B). Among children 24–59 months of age, the housefly population density spike in 2009 was followed by a surge in *Shigella*-associated MSD cases approximately one month later, while the housefly spike in 2010 was followed by a surge in *Shigella*-associated MSD cases about two months later (Figure 3C).

### GLM Poisson time series model

**Figure 5:**
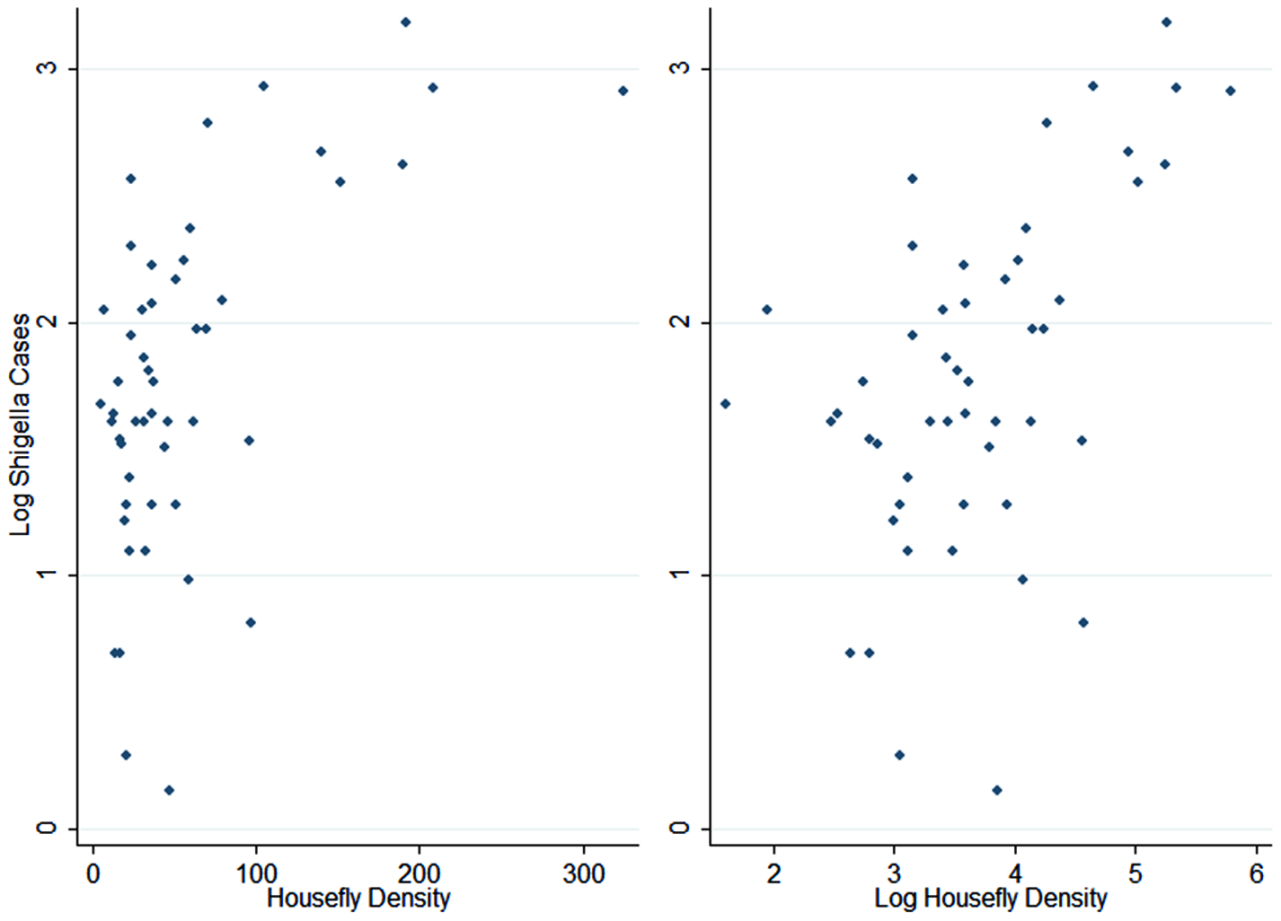
Scatterplot of log *Shigella* case counts against fly density untransformed (left) and log scale (right).

The log scale was found to be more appropriate than the untransformed scale for the lagged fly counts (Figure 5). The best fitting Poisson model used *Shigella* case counts in the log scale at a lag of one biweekly period to account for autocorrelation (Tables 2–3). Log housefly population density was positively associated with *Shigella* case counts at a three-period temporal lag, (Table 3). Each log increase of houseflies was associated with an IRR of 1.39 three periods later (95%CI: 1.23 to 1.58).

**Table 2:** GLM Poisson regressions of *Shigella* case counts on lags of log fly counts.*

Housefly count lag	Incidence Rate Ratio	Standard Error	p-value	95% CI	AIC **	BIC **
Log fly count (0 lags)	1.18	0.07	<0.01	1.05 to 1.34	252.8	258.3
Log fly count (1 lags)	1.25	0.08	<0.01	1.10 to 1.41	247.5	253.0
Log fly count (2 lags)	1.30	0.08	<0.01	1.16 to 1.47	242.4	247.8
Log fly count (3 lags)	1.39	0.09	<0.01	1.23 to 1.58	235.2	240.7
Log fly count (4 lags)	1.39	0.10	<0.01	1.20 to 1.61	242.1	247.5
Log fly count (5 lags)	1.30	0.10	<0.01	1.12 to 1.52	249.5	255.0
Log fly count (6 lags)	1.10	0.08	0.21	0.95 to 1.28	259.9	265.4
Log fly count (7 lags)	1.01	0.07	0.90	0.88 to 1.16	261.4	266.9

* Autocorrelation accounted for by regression on 1 lag of *Shigella* case counts.

** Obtained on n-7 sample size (as with 7 lags) for comparability between models.

The above table shows GLM Poisson regressions performed with no lag, then every lag up to 7 lags, among children 12 to 59 months of age.

**Table 3:** GLM Poisson time series model of *Shigella* case counts on log housefly count.

Accounting for auto-correlation	Controlling for temperature	Variable	β	Standard Error	p-value	Incidence Rate Ratio	95% CI
No	No	Log fly count (3 lags)	0.48	0.06	<0.01	1.61	1.44 to 1.81
		Intercept	0.15	0.24	0.52	-	-
Yes	No	Log fly count (3 lags)	0.33	0.07	<0.01	1.39	1.23 to 1.58
		Log *Shigella* case count (1 lag)	0.42	0.09	<0.01	-	-
		Intercept	-0.09	0.24	0.71	-	-
Yes	Yes	Log fly count (3 lags)	0.32	0.06	<0.01	1.37	1.21 to 1.56
		Log temperature (4 lags)	1.28	0.47	<0.01	3.60	1.42 to 9.10
		Log *Shigella* case count (1 lag)	0.32	0.09	<0.01	-	-
		Intercept	−4.04	1.50	<0.01	-	-

### Temperature as a potential confounder

As the air warmed in springtime to temperatures favorable for housefly reproduction (minimum temperatures above 20C [27]), housefly population density increased both in 2009 and 2010 (Figure 4). As the air continued to warm into the summertime to temperatures that favor growth of *Shigella* spp. (maximum temperatures approaching 37°C) [28], [29], *Shigella*-positive case counts were observed to increase in 2009 and 2010 (Figure 4). Because temperature may have been responsible for the association between housefly population density and *Shigella* (either completely, or in part), we explored average temperature as a potential confounder. As with fly counts, the log scale was found to be more appropriate than the untransformed scale for mean temperature. A GLM Poisson model with *Shigella* case counts as the outcome, accounting for autocorrelation by including a variable for *Shigella* case counts in the logarithmic scale at one lag, showed that each log increase in mean temperature was associated with an IRR of 4.09 (95% CI: 1.70 to 9.87) four periods later. When added to the model that included log housefly population density at three lags, log average temperature at four lags resulted in the best model fit. However, the association between log housefly population density and Shigella case counts was essentially unchanged (IRR = 1.37, 95% CI: 1.21 to 1.56) (Table 3). As there was no evidence of confounding, mean temperature was not included in the final model.

### Autocorrelation-adjusted attributable fraction

Among children <5 years of age, if housefly population density were diminished to the average level of fly count in the lowest decile, an intervention might have prevented 37.4% (95% CI: 16.9 to 57.9) of the total *Shigella*-associated MSD cases (Table 4). If housefly population density were diminished to the level observed in the lowest 3 deciles, an intervention might have prevented 29.7% (95% CI: 12.9 to 46.6). Reducing housefly population density to the level observed in the lowest 5 deciles might have prevented 26.1% (95%CI: 14.1 to 38.1) of *Shigella*-associated MSD cases.

**Table 4:** Procedure for calculating the autocorellation-adjusted attributable fraction (AF) using the Bruzzi procedure.

Decile	Log fly count	*Shigella* cases	IRR	AF among cases [(IRR-1)/IRR]	Attributable cases
1	2.24	25.25	1	0	0
2	2.84	20.45	1.096	0.088	1.79
3	3.11	41.93	1.201	0.167	7.02
4	3.38	19.21	1.316	0.240	4.62
5	3.52	26.94	1.443	0.307	8.27
6	3.69	28.41	1.581	0.368	10.44
7	3.96	25.62	1.733	0.423	10.83
8	4.18	39.10	1.899	0.474	18.52
9	4.62	48.07	2.082	0.520	24.98
10	5.32	87.66	2.281	0.562	49.24
	**Totals**	362.64			135.71

Attributable Fraction (AF) = 0.374.

Sample variance (50 iteration jackknife) = 0.0002273.

Jackknife variance of AF [(sample variace*(n−1)^2^)/n] = .01091495.

95% CI of AF: = 0.169 to 0.579.

The AF was based on IRRs calculated for each decile of fly count relative to the first decile (referent) and adjusted for autocorrelation using the Bruzzi procedure, with variance calculated using a jackknife procedure.

## Discussion

The epidemiologic behavior of *Shigella* infections has fascinated and perplexed epidemiologists and microbiologists for many years. Recognition of the minuscule infectious dose of *Shigella* (ten colony forming units) [1], [2] that can cause full blown clinical disease explains its transmission by direct fecal-oral contact, its propensity to be spread in sub-populations even in industrialized countries if personal hygiene is compromised, and underlies the propagated epidemic pattern observed in shigellosis outbreaks [30]. Two notable features of *Shigella* disease in developing countries are its seasonality and its temporal association with houseflies [7]. It has long been recognized that a marked increase in *Shigella* dysentery cases accompanies or follows shortly after the annual seasonal increase in the density of houseflies. This association has been noted in tropical [12], sub-tropical [11] and temperate [31] regions of the world. The Mirzapur GEMS site offered an opportunity to investigate in depth the association of shigellosis in relation to housefly density. This paper reports results of applying the appropriate time-series analysis to these unique entomological, clinical and microbiologic datasets.

Housefly population density in Mirzapur peaked in February and March of 2009 and 2010 (Figures 3A–C), indicating an annual “fly season”. Housefly densities vary with temperature (20–25°C is most favorable), number of sunshine hours, humidity and availability of breeding sites [27]. In tropical and subtropical climes, fly density increases as mean daily temperature rises following the end of the cool season; however, as mean daily temperatures approach their peak in the hot season, housefly density then decreases. Reports from elsewhere in South and Southeast Asia have also identified marked fly seasons in the springtime before the full heat of summer, as in Uttar Pradesh, India (fly density peak in February and March) [32], North West Frontier Province, Pakistan (peak in March–June) [11], and central Thailand (March–June) [12].

*Shigella*-positive acute MSD cases also showed a marked seasonality in Mirzapur, with surges occurring in the summer months of March–June 2009 and March–July 2010 (Figures 3 and 4), when air temperature nears the 37°C optimum for growth of *Shigella* bacteria is [28], [29]. Once again, multiple reports from Asia have similarly noted an April–May surge in *Shigella* infections, as in Dhaka [33], central Thailand [34] and Jakarta, Indonesia, indicating a regional phenomenon in areas with similar climates.

It is well-recognized that the incidence of shigellosis is much higher in children 12–48 months of age than in infants 0–11 months of age [30], [35]. Accordingly, in Mirzapur, the peak of housefly density that was followed six weeks later by a surge in *Shigella*-associated MSD cases was seen among children 12–59 months of age (Figure 4), showing a strong, statistically significant association (Tables 2–3).

The shape of the spikes in fly density also corresponded well with the subsequent surges in *Shigella*-associated case presentations, further suggesting a causal association. During World War 1, Dudgeon observed in Macedonia that a spike in housefly density in April–May was followed one month later by a spike in *Shigella* incidence in British Army field hospitals [36]. In Mesopotamia between July 1916 and December 1918, Ledingham also noted April–May surges in fly density that were followed two weeks to one month later by an increased incidence of dysentery [37]. Ledingham proposed an explanation for this delay that could also apply to the young children in Mirzapur. He suggested that the springtime surge in fly density leads to an abundance of mechanical vectors capable of contaminating food and cooking and eating utensils with *Shigella*. Subsequent ingestion of the contaminated food or handling of the contaminated fomites (eating utensils) by susceptibles thereupon establishes many new *Shigella* infections. This initial burst of *Shigella* infections that shortly follows the peak fly density results in a temporary surge in the magnitude of the human reservoir of *Shigella* from which transmission then ensues by more usual modes during the hot summer months, in particular by direct contact transmission.

Because a housefly's habitat can range over a two-mile radius [7], [38], from a few foci where the flies encounter human feces containing *Shigella*, they can thereupon effectively “seed” a much broader and more dispersed human population with *Shigella*, as the flies alight on human food and eating utensils. The highly transmissible *Shigella* can then continue to spread through person-to-person (and occasional foodborne) transmission within families [39] and across wider geographic areas [40]. Indeed, our GLM Poisson time-series model showed precisely this effect – i.e., housefly population density was associated with *Shigella* MSD three periods (six weeks) later (Table 3), an association that was not confounded by mean ambient temperature. This suggests that houseflies may be seeding the population with *Shigella* infections, resulting in many small outbreaks at about a six-week lag. The noise inherent in these data does not preclude the possibility of associations occurring at multiple lags, simultaneously. Indeed,*Shigella*-associated MSD cases appeared to be associated with fly density at several lags, but only a lag of three periods was retained in a model when multiple lags were included together (Table 3). We note also that the logarithmic nature of the association between fly density and log *Shigella* case counts suggests a biological process (Figure 5).

The AF calculation allowed us to estimate the potential effect of a public health intervention that was highly successful, eliminating fly density peaks by reducing housefly density to a very low level (the average in the lowest decile) (Table 4). We also estimated the effect of a less highly successful intervention (reducing fly density to the average in the 3 lowest deciles) and moderately successful intervention (reducing fly density to the average in the lowest 5 deciles). If a highly successful intervention could be instituted in a setting such as Mirzapur, it might prevent approximately 37% of the *Shigella* cases observed over the study period, assuming a causal association. More rigorous interventions that decreased fly density to an even lower level presumably might achieve even greater efficacy. A less highly successful intervention might prevent 30% of the *Shigella* cases observed, while a moderately successful intervention might prevent 26%, showing that an intervention might produce robust results even for moderate reductions in housefly density. Assuming the association between housefly density and *Shigella* infection is causal, this means fly control could potentially rank highly among other public health interventions as a means of preventing shigellosis (and perhaps other diarrheal infections such as those caused by enterotoxigenic *Escherichia coli*) [6].

Several limitations should be taken into account when interpreting the results of this study: 1) We assume that children seen at sentinel health centers are representative of all children in the DSS population. However, children seen at the health centers may be subtly different from children in the community whose families do not take them to health centers when they have diarrhea. 2) The use of a limited number of sentinel households where fly density was measured that were clustered in the most highly populated area of Mirzapur may not have been optimal for measuring a site-wide fly density value, and certainly it did not enable analysis by geographic area. However, one may argue that the wide housefly flight radius [7], [38] means that a limited number of surveillance sites may be used to represent flies as if they are a site-wide environmental exposure, as with studies of particulate pollution that often use a single site for their exposure measurements [41]. 3) Lastly, though we found that temperature was not a confounder, the presence of other unknown confounding factors could have resulted in some bias in our estimates.

Baited fly trap technology constitutes one inexpensive, effective tool for reducing housefly density, when implemented as part of a well-designed fly mitigation strategy [6], [7]. Moreover, manufacture of simple fly traps could become a local cottage industry [42], [43]. Whereas the importance of fly control in reducing the incidence of pediatric diarrhea and dysentery was recognized in the past [14], [15], [19], [31], [36], [37], the modern public health community has not generally embraced fly control efforts as a public health imperative. Our experience instructs that this is largely based on the lack of familiarity with information about the role of flies in the transmission of *Shigella* (and perhaps other enteric pathogens) and a lack of knowledge of of baited fly traps as an effective, affordable, environmentally-friendly measure to reduce housefly density. The time is ripe for a modern, cluster-randomized trial that can not only establish unequivocally whether a causal relationship exists between houseflies and *Shigella* transmission but can also quantify the effectiveness of baited fly traps (alone or in conjunction with other interventions that decrease fly density) on diminishing *Shigella* disease.
